# Zinc Binding Properties of Engineered RING Finger Domain of Arkadia E3 Ubiquitin Ligase

**DOI:** 10.1155/2010/323152

**Published:** 2010-06-27

**Authors:** Christos T. Chasapis, Ariadni K. Loutsidou, Malvina G. Orkoula, Georgios A. Spyroulias

**Affiliations:** Department of Pharmacy, University of Patras, Panepistimioupoli, Rion, 26504 Patras, Greece

## Abstract

Human Arkadia is a nuclear protein consisted of 989 amino acid residues, with a characteristic RING domain in its C-terminus. The RING domain harbours the E3 ubiquitin ligase activity needed by Arkadia to ubiquitinate its substrates such as negative regulators of TGF-*β* signaling. The RING finger domain of Arkadia is a RING-H2 type and its structure and stability is strongly dependent on the presence of two bound Zn(II) ions attached to the protein frame through a defined Cys3-His2-Cys3 motif. In the present paper we transform the RING-H2 type of Arkadia finger domain to nonnative RING sequence, substituting the zinc-binding residues Cys^955^ or His^960^ to Arginine, through site-directed mutagenesis. The recombinant expression, in *Escherichia coli*, of the mutants C955R and H960R reveal significant lower yield in respect with the native polypeptide of Arkadia RING-H2 finger domain. In particular, only the C955R mutant exhibits expression yield sufficient for recombinant protein isolation and preliminary studies. Atomic absorption measurements and preliminary NMR data analysis reveal that the C955R point mutation in the RING Finger domain of Arkadia diminishes dramatically the zinc binding affinity, leading to the breakdown of the global structural integrity of the RING construct.

## 1. Introduction

**Figure 1:**
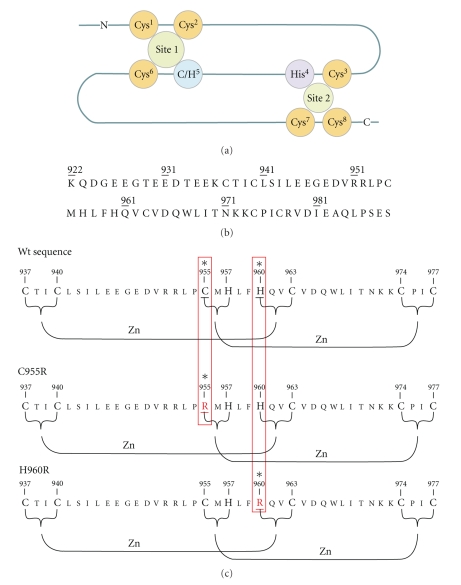
(a) The schematic structure of a RING finger domain showing the “cross-brace” arrangement of the two zinc binding sites; (b) The amino acid sequence of the RING finger domain of mouse Arkadia; (c) the mutation sites, C955R and H960R, in the sequence of Arkadia RING Finger domain.

RING finger is a characteristic protein sequence motif that was first identified in the protein product of the human gene *RING1*—*Really Interesting New Gene 1*—which is located proximal to the major histocompatibility region on chromosome 6 [1]. The RING finger domain is a cysteine/histidine-rich, zinc-chelating domain that promotes both protein-protein and protein-DNA interactions [2]. It is defined as Cys^1^-Xaa_2_-Cys^2^-Xaa_9–39_-Cys^3^-Xaa_1–3_-His^4^-Xaa_2-3_-Cys/His^5^-Xaa_2_-Cys^6^-Xaa_4–48_-Cys^7^-Xaa_2_-Cys^8^ (where Xaa can be any amino acid residue). Two zinc atoms are complexed by the cysteine/histidine residues in a “cross-brace” manner, to provide correct folding and biological activity to the RING domain Figure 1(a) [3, 4]. The distinctive cross-brace arrangement of the two tetrahedral zinc binding sites endows the RING domain with a globular conformation, characterized by a central *α*-helix and variable-length loops separated by several small *β*-strands. RING finger motifs are further subdivided, depending on whether a cysteine or histidine residue is found at Cys/His^5^ within the motif. Thus they are classified as being either a RING-HC (Cys^5^) or a RING-H2 (His^5^) type. The metal binding properties of some RING finger domains have been investigated in a preliminary manner, using synthetic peptides that bear the amino acid sequence of BRCA1 and HDM2 [5, 6]. It is well-established that RING fingers can also bind other than zinc metal ions, such as cobalt (II) and cadmium (II) [7–9] 

Human Arkadia is a nuclear protein of 994 amino acid open reading frame (ORF) that exhibits no sequence similarity with any other known protein apart from the RING-H2 finger topology in its C-terminal region [10]. Mouse Arkadia ORF differs in length by 5 residues (989 aa) but their C-terminal 124 residue segments bear identical amino acid sequence. Arkadia has been shown to function as an E3 ubiquitin ligase [11]. E3 ligases participate actively at the last step of the protein degradation through the ubiquitination pathway [12, 13]. Additionally, RING E3 ubiquitin ligases play an essential role in the regulation of many biological processes and defects in some of them are involved in cancer development. Thus, RING E3 ligases represent a potential molecular target for disease intervention in mechanism-driven drug discovery [14–17]. Arkadia is the first example of a RING E3 ubiquitin ligase that positively regulates TGF-*β* family signaling. The RING-H2 domain harbours the E3 ubquitin ligase activity required by Arkadia to ubiquitinate various negative regulators of TGF-*β* [11, 18–20]. Recently an efficient method for expression in *Escherichia coli* and purification of recombinant Arkadia RING-H2 finger domain as a soluble protein was reported [21]. Preliminary NMR data analysis of the Arkadia RING Finger suggests a *β* *β* *α* *β*-like topology typical of RING finger domains. Additionally, NMR experimental evidence indicates that proper zinc incorporation is a major determinant for the RING finger domain structure and stability [21]. 

Each type of RING domain exhibits unique structural characteristics, which may affect the specificity of the recruitment of E2 enzymes or substrate recognition. Furthermore, substitution of various native amino acids, especially the zinc-binding residues, resulted in the significant loss of ubiquitination capacity, as shown in various E3 ligases such as BRCA1, HDM2 and many other RING finger domain-containing proteins. Substitution of zinc-binding amino acids with residues by atom-donors with lower affinity to zinc metal ion, results in a collapsed RING finger structure or in a conformational alteration that prevents RING from interacting with E2. Thus assembly of E2 and E3 into a productive complex, a key component in the ubiquitination pathway, is misformed. Herein we present the production of two nonnative RING finger domains of mouse Arkadia: C955R and H960R Figure 1(b), through site-directed mutagenesis. The recombinant expression in *Escherichia coli* of the mutants C955R and H960R reveals significant lower yield in respect with the native polypeptide of Arkadia RING-H2 finger domain. Only the C955R mutant exhibits expression yield sufficient for recombinant protein isolation and the protocol applied was based on that used for the wt of Arkadia RING-H2 finger domain [21]. Since zinc binding is considered to be critical to proper folding and activity of the RING-H2 domain of Arkadia, the nonnative polypeptides were expressed in zinc-loaded growth media. Zinc contents of the wt and mutant C955R RING construct were determined by Atomic Absorption measurements. Finally, the folding state and the structural integrity of the holo-RING constructs were monitored through ^1^H and 2D ^1^H-^15^N-HSQC spectroscopy.

## 2. Materials and Methods

### 2.1. Plasmids, Bacterial Strains, and Growth Media

*E. coli* DH5*α* (Invitrogen) was used as the host strain for cloning and plasmid propagation and *E. coli* BL21(DE3) (Stratagene) as the host strain for the expression vector pGEX–4T-1 (Amersham Biosciences). Both strains were routinely grown in Luria-Bertani (LB hereafter) broth or on plates of LB agar, supplemented with ampicillin (100 *μ*g/ml) for both transformed DH5*α* and BL21(DE3) strains. Induction was carried out in M9 medium supplemented with 2 ml/l of solution Q (40 mM HCl, 50 mg/l FeCl_2_·4H_2_O, 184 mg/l CaCl_2_·2H_2_O, 64 mg/l H_3_BO_3_, 18 mg/l CoCl_2_·6H_2_O, 340 mg/l ZnCl_2_, 605 mg/l Na_2_Mo_4_·2H_2_O, 40 mg/l MnCl_2_·4H_2_O) and 10 ml/l of vitamin mix (500 mg/l thiamine, 100 mg/l biotin, 100 mg/l choline chloride, 100 mg/l folic acid, 100 mg/l niacinamide, 100 mg/l pantothenic acid, 100 mg/l pyridoxal, 10 mg/l riboflavin).

### 2.2. Cloning, Expression, Purification, and Isotopic Labeling of Arkadia RING Constructs

The protocol adopted to express and purify the nonnative RING constructs of Arkadia was essentially the same as that previously reported to produce the wt of the RING-H2 construct [10]. The expression plasmids for the presented mutants were obtained from that of wt RING-H2 through the Quickchange (Stratagene) mutagenesis kit. ^15^N isotopic labeling of the mutant C955R was obtained using M9 medium supplemented with 2 g/l ^15^NH_4_Cl as nitrogen source.

### 2.3. Preliminary Protein Characterization

The purity of all polypeptides was checked by SDS-PAGE in 17% polyacrylamide gels after staining of protein bands with Coomassie Blue R-250. The concentration of RING domains of Arkadia was determined by using two methods: the first is based on the extinction coefficients at 280 nm of each construct, as estimated by PROTPARAM software (http://http://www.expasy.org/tools/protparam.html/) and the second, which is more accurate, on the Quick start Bradford protein assay (BIORAD) [22].

### 2.4. Atomic Absorption Spectrometry

Elemental analysis for zinc was performed employing Atomic Absorption Spectrometry (Perkin Elmer AAnalyst 300). The zinc content was measured in wt RING-H2 and the mutant C955R RING constructs. In order to exclude any matrix effect in zinc measurements, the standard addition method was applied [23]. Four samples were prepared for the first construct, the unspiked and three aliquots (spiked) to which known amounts of the analyte zinc from a stock standard solution (AA Panreac 1.000 ± 0.002 g/l Zn) were added (Table 1). For the nonnative construct, three samples were prepared, respectively: the unspiked and two spiked. The spiked concentration ranged from 0 (unspiked sample) to 0.6 ppm. The volume was kept equal for all samples by means of addition of PBS buffer. Preliminary tests assured that the overall concentration of zinc was lying within the linear range of the technique (1 ppm). Absorbance of all samples at the appropriate wavelength (213.9 nm) was measured twice. An appropriate calibration curve for each protein was constructed (Table 3, Figure 3). The concentration of zinc in the unspiked samples was determined, dividing the intercept by the slope of calibration curve [23].

### 2.5. NMR Spectroscopy

1D ^1^H-NMR and ^1^H-^15^N HSQC spectra were recorded at 298 K on a Bruker Avance 600 MHz spectrometer, equipped with a cryogenically cooled pulsed-field gradient triple-resonance probe (TXI) and on a Bruker Avance DRX 400 MHz, equipped with two multinuclear broadband probes (inverse BBI and direct BBO). ^1^H 1D spectra were acquired in the 16 ppm spectral width using a variety of pulse sequence for water suppression and spectra were calibrated relatively to the water proton resonances. The 2D ^15^N HSQC spectra were acquired using a spectral width of 40 ppm for *ω*1 and 8 ppm for *ω*2. The NMR samples contained 1 mM and 0.2 mM protein of wt Arkadia and mutant construct C955R, respectively, in 50 mM phosphate buffer, pH = 7, in the presence of 90% H_2_O/10% D_2_O.

## 3. Results and Discussion

**Figure 2:**
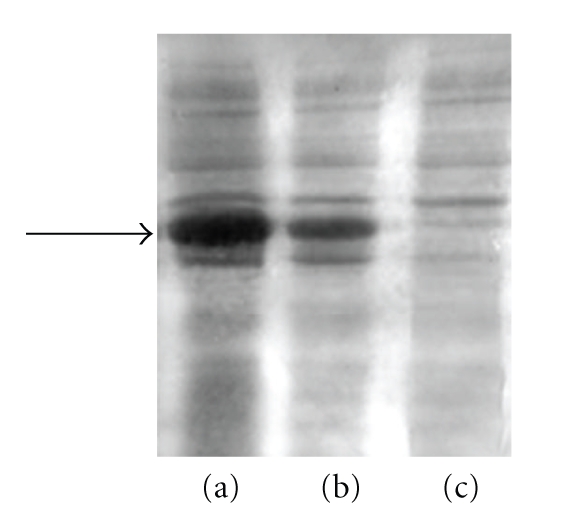
The total cell lysate of wt (a), the mutant C955R (b), and H960R (c) at 37°C with ZnCl_2_ at a final concentration 100 *μ*M.

The C-terminal RING finger domains of wt and Arkadia mutants were expressed in M9 minimal growth media supplemented with ZnCl_2_. Both of the mutants: C955R and H960R, reveal remarkably lower expression yield in respect with the native polypeptide of Arkadia RING-H2 finger domain. Small-scale test expression of RING constructs in the absence of ZnCl_2_ suggests that additional zinc enrichment of growth media does not affect the expression yield of soluble RING constructs. In Figure 2, it is presented the total cell lysate of wt (a), the mutant C955R (b) and H960R (c), at 37°C with ZnCl_2_ at a final concentration 100 *μ*M. Finally, from the above mutants, the expression yield of the C955R mutant was sufficient for recombinant protein isolation and preliminary studies.

**Figure 3:**
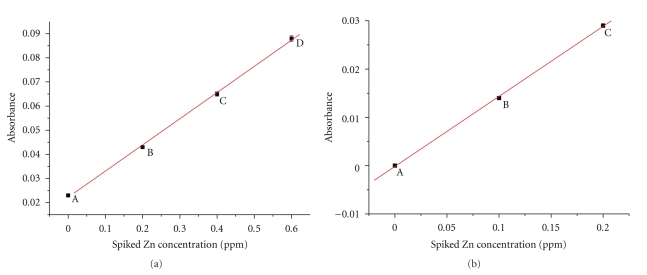
Calibration curves for the additions of zinc in (a) RING-H2 and (b) mutant RING (C955R) of Arkadia.

Elemental analysis for zinc was performed employing Atomic Absorption Spectrometry. A calibration curve for each protein sample (Figure 3) was constructed as described in Section 2. The concentration of zinc in the unspiked sample of both constructs was determined, dividing the intercept by the slope of the calibration curves [23] (Table 3). The concentration of zinc in the initial samples of 0.5 mM RING-H2 and 0,2 mM C955R mutant of Arkadia was calculated to be 1 mM and 0 mM, respectively, (Table 2). It should, also, be stressed that the zinc content of the constructs remained stable in excess of ZnCl_2_ in the growth media (>100 *μ*M). The atomic absorption measurements of the native RING construct suggest a stoichiometry of two zinc ions per molecule, a value that is in agreement with the classic binding properties of RING fingers. However, the elemental analysis of the C955R mutant suggests that the mutated construct does not bind zinc at all, indicating that the substitution of one of the three cysteines in the second tetrahedral zinc binding motif abolishes the global metal binding capacity of the hole polypeptide. 

**Figure 4:**
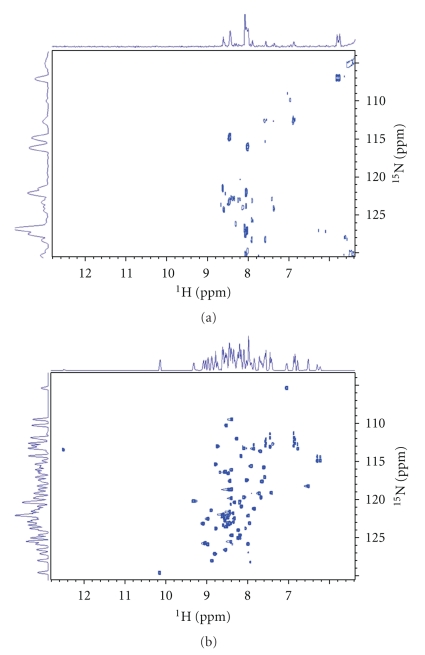
The 1D ^1^H-NMR of amide proton resonances (7,5–10 ppm) and ^1^H-^15^N HSQC of the mutant (a) and the native RING (b).

It is known that the protein structure of the Arkadia zinc loaded wt RING-H2 domain is well folded and the zinc incorporation is a major determinant for its structure and stability [17–21]. Indeed, when EDTA was added to a sample of the Arkadia RING finger domain at a 1 : 1 molar ratio with respect to Zn(II) substantial changes were observed for a number of residues in a ^1^H-^15^N HSQC spectrum. Further addition of EDTA up to a final Zn : EDTA molar ratio of 1 : 2 led to dramatic changes in the ^ 1^H-^15^N HSQC typical for structureless polypeptide [21]. The 1D ^1^H-NMR spectrum of C955R mutant (Figure 4(a)) and its comparison with the 1D NMR spectrum of the wt (Figure 4(b)) suggests that the C955R RING construct is not a folded protein. 

Heteronuclear NMR spectroscopy, and especially 2D ^1^H-^15^N-HSQC experiments provides the fingerprint spectrum of each polypeptide and are diagnostic tools for the folding properties and aggregation state of recombinant proteins. Additionally, this technique can probe fine structural rearrangements upon amino acids substitutions and for this reason it is applied to probe the effect of C955R mutation. Indeed, the ^1^H-^15^N-HSQC exhibits dramatic changes in respect with the spectrum of the native RING construct, with no signal dispersion of the backbone amide groups (Figure 4). This remarkable lack of the chemical shift dispersion is almost identical to that observed in the HSQC spectrum of the unstructured wt RING construct, due to the presence of EDTA excess [21]. EDTA is a powerful metal chelating agent and competes the RING polypeptide for zinc binding. Excess of EDTA in RING Finger solution uptakes the zinc ion and RING polypeptide structure collapses as evidenced by the HSQC. Mutation of the Cys^955^ by Arg seems to result to an unstructured polypeptide that has abolished the zinc binding ability. 

The substitution of the His^960^ and Cys^955^ residues by an Arg was driven by the fact that Arg, according to Blosum matrix, is among the residues with the higher probability to substitute His in polypeptide sequence, due to the similar length and nature of its side chain and its basic character. Additionally, its linear side-chain is not expected to impose significant steric interaction that may alter dramatically the spatial geometry of the other potential Zn(II) binding residues (three Cysteines). Since the expression yield of the H960R mutant was very low for protein isolation, preliminary studies were performed only for the C955R mutant. It is proposed that the new mutated motif does not bind zinc at all, in contrast to the stoichiometry of two zinc ions per molecule for the native RING construct. Although the alteration occurred in the second binding motif, both binding sites are losing their metal binding capacity. Additionally, NMR spectroscopy indicates that C955R mutant of RING Arkadia undergoes significant structural rearrangements losing its proper folding, in respect with the native polypeptide. This result may provide new insights into the sequential metal loading by the two tetrahedral binding sites of the RING-H2 domain of Arkadia. Since the net effect of the C955R substitution is the collapse of the structure it is suggested that the second site may be an important regulator that affects the formation of the first site and the overall metal-dependent folding of the polypeptide. In the past, the sequential binding was considered to be a general characteristic of RING finger domains, but its biological significance has not yet been established [5, 24, 25]. One characteristic example is the metal binding properties of peptide corresponding to the RING finger domain from the tumor suppressor gene product BRCA1. It is found that the above metal binding is thermodynamically *sequential *with cobalt(II) almost saturating one of the two sites in each polypeptide prior to binding to the other site. Analysis of the absorption spectra due to cobalt(II) bound to the two sites revealed that the higher affinity site is comprised of four cysteinate ligands whereas the lower affinity site has three cysteinate and one histidine ligands. Upon binding a metal ion to site 1 (Cys4), the peptide becomes somewhat more structured and, perhaps, more rigid in a manner that affects the regions involved in forming site 2 (Cys3His). This, in turn, increases the difficulty in binding a metal ion to site 2, leading to the observed anticooperativity [5]. The same metal binding properties were found in the RING finger domain of HDM2 C-terminal domain. In this case the metal binds to the higher-affinity binding site were monitored through fluorescence energy transfer, revealing two binding events separately: the stronger binding site C4 and the weaker one C3H. Furthermore, synthetic knockout mutants not only enabled to assign the coordinating residues in the HDM2 RING finger domain but also revealed that metal binding to HDM2 was also anticooperative [6]. 

In summary, we reinforce the theory that substitution of zinc-binding amino acids in RING finger domains may possesses an immense role in the biochemical and biological activity of RING Finger E3 ligases. Herein, we demonstrate that even one point mutation (C955R) in the RING finger domain of Arkadia can have a dramatic effect in the domain structure, leading to the breakdown of the global structural integrity of the RING construct. This phenomenon is expected to prevent the formation of a E3-E2 productive complex and finally to reduce its ubiquitin ligase activity. In the past, similar results have been reported regarding mutants in the zinc binding motif of HDM2 (429–491). RING finger domain (such as His452Ala and His457Ala) was studied through NMR spectroscopy revealing a lack of dispersion and broad resonances in ^15^N-HSQC spectra, characteristic of unfolded protein [26]. The correlation between mutations in the RING finger domain of E3 ligases and their ubiquitination activity was well established in the past through the detection of mutations within the BRCA1 RING domain that predispose to cancer due to the abolishment of BRCA1 ligase activity [27]. Furthermore, site mutations in the RING finger of E3 ligases are associated with various consequences in biological processes such as their unusual distribution in the cell, like the large cytoplasmic and nuclear inclusion of the two mutant isoforms of Parkin RING finger protein [28]. In this light, the verification of this probable occurrence of a sequential metal binding by the wt/mutant of RING finger domains of Arkadia and the study of their mechanism remain to be done. 

## Figures and Tables

**Table 1:** Samples of each construct spiked with Zn prepared and analyzed.

Sample	Volume of the unknown protein sample used (*μ*l)	Final volume of the sample (ml)	Spiked Concentration of Zn (ppm)
*RING-H2 construct*			
A	15.0	5.0	0
B	15.0	5.0	0.2
C	15.0	5.0	0.4
D	15.0	5.0	0.6
*Mutant RING construct (C955R)*			
A	9.0	2.0	0
B	9.0	2.0	0.1
C	9.0	2.0	0.2

**Table 2:** Cumulative results of zinc : protein ratio for the samples analyzed.

Protein sample	Zn concentration (mM)	Protein concentration (mM)	Zn: protein ratio
RING-H2	1.04300 (±0.00460)	0.498 (±0.001)	2.094
Mutant RING (C955R)	0	0.201 (±0.001)	0

**Table 3:** Linear regression data for the calibration curves.

Protein sample	Intercept (A)	Slope (B)	Correlation Coefficient (R)
RING-H2	0.02220 (±0.00009)	0.10850 (±0.00240)	0.99951 (±0.00107)
Mutant-RING (C955R)	−0.00002 (±0.00004)	0.14500 (±0.00289)	0.99980 (±0.00004)

## Abbreviations

RING: Really interesting new gene
TGF-*β:*: Transforming growth factor *β*
NMR: Nuclear Magnetic Resonance.
